# The mitochondrial genome of *Tipula* (*Vestiplex*) *aestiva* Savchenko, 1960 (Diptera: Tipulidae)

**DOI:** 10.1080/23802359.2023.2172975

**Published:** 2023-02-05

**Authors:** Yuetian Gao, Bing Zhang, Ding Yang, Yan Li

**Affiliations:** a Department of Entomology, College of Plant Protection, China Agricultural University, Beijing, P.R. China; b Key Laboratory of Economic and Applied Entomology of Liaoning Province, College of Plant Protection, Shenyang Agricultural University, Shenyang, Liaoning, P.R. China

**Keywords:** Tipulidae, *Tipula*, mitochondrial DNA, phylogenetic analysis

## Abstract

The subgenus *Vestiplex* of the genus *Tipula* is one of the most species-rich groups in the family Tipulidae. Here, we present the first mitochondrial genome of the subgenus *Vestiplex.* The nearly complete mitochondrial genome of *Tipula aestiva* Savchenko, 1960 (Genbank accession number: OM287601) was 16083 bp in length and consisted of 13 PCGs, 22 tRNA and two rRNA genes. The phylogenetic the tree of family Tipulidae was reconstructed based on 13 PCGs sequences using the maximum likelihood method. The result strongly supported the monophyly of the family Tipulidae. The subgenus *Vestiplex* is indicated as the sister group of subgenera *Pterelachisus* and *Formotipula*.

## Introduction

The genus *Tipula* is one of the most diverse groups in the family Tipulidae with over 40 subgenera and 2400 species, which are distributed worldwide (Oosterbroek 2022). However, the monophyly of the genus has long been a controversial issue. Since the mitochondrial (mt) genomes were introduced into phylogenetic studies, several *Tipula* species of different subgenera had their mitogenomes sequenced and published, including those of subgenera *Acutipula*, *Dendrotipula*, *Formotipula* (Zhang et al. 2019), *Lunatipula*, *Nippotipula* (Beckenbach 2011), *Pterelachisus*, and *Yamatotipula* (Zhao et al. 2019). As one of the largest subgenera of the genus *Tipula*, *Vestiplex* contains over 180 described species worldwide, but none of which had its mitogenome available. Adults of *Vestiplex* usually occur in forests or grasslands, while their larvae were considered to be saprophagous (Gelhaus 1986). Adults can generally be characterized by the medium-sized body with the patterned wings and the ninth tergite with a shallow concavity and sclerotized saucer or completely divided longitudinally by the pale membrane (Alexander 1935; Alexander and Byers 1981) and larvae can generally be characterized by the dark band separating anus from anal papillae, in addition to marginal band encircling the anal area (Gelhaus 1986). Here, we presented the nearly complete mitochondrial genome of *Tipula aestiva* Savchenko, 1960, which is the first mitochondrial genome of the subgenus *Vestiplex*. The results provide valuable information for future phylogenetic and evolutionary studies.

## Materials and methods

The specimen of *Tipula* (*Vestiplex*) *aestiva* Savchenko, 1960 (Voucher number: CAUGS20200802) were collected by Shang Gao on 2 August 2020 from Zhamashixigou (38.2139 N, 100.0368E, 3048 m), Qilian, Qinghai Province, China. It was preserved in 95% ethanol and deposited in the Entomological Museum of China Agricultural University, Beijing (Liang Wang, 1352659341@qq.com). The specimen were identified by Yuetian Gao based on the following morphological characteristics: flagellomeres with verticils shorter than half of corresponding segments; tergite 9 in the shape of the slightly concaved saucer, with the elevated anterior border (Starkevich et al. 2019). Whole genomic DNA was extracted from the thorax of a single specimen using QIAamp DNA Blood Mini Kit (Qiagen, Germany). Then, the DNA concentration was measured by Agilent 5400. The sequencing library was generated using NEB Next® Ultra™ DNA Library Prep Kit. The sample was subsequently sequenced paired-end on one lane with Illumina NovaSeq 6000, with the desired insert size of 350 bp and read length of 150 bp. Approximately 6 G of raw data was produced by sequencing. The processes above were accomplished by the Novogene biotechnology company (Beijing). The mitochondrial genome was assembled and annotated by MitoZ (v.2.3). And the start and stop codons of each protein-coding gene (PCG) were checked manually in Geneious (v. 9.0.2).

## Results

The nearly complete mitogenome of *Tipula* (*Vestiplex*) *aestiva* was 16,083 bp in length (Genbank accession number: OM287601), including 13 protein-coding genes, 22 tRNA genes and 2 rRNA genes (12S rRNA and 16S rRNA). The arrangement of all mitochondrial genes matched other published mitogenomes of Tipulidae species (Zhang et al. 2019; Beckenbach 2011; Zhao et al. 2019). The control region was incompletely obtained in this research. The nucleotide composition of the mitogenome was biased toward A and T, with 77.4% of A + T content (*A* = 38.9%, *T* = 38.5%, *G* = 8.9%, *C* = 13.7%). Most PCG genes start with ATT, ATG or ATA codons, except that three genes (COI, NAD5 and NAD1) start with TCG, CAC, CAA codons, respectively. Most PCG genes stop with TAA, TAG or TAT codons, but NAD4L stops with ATT codons. Two PCG genes stopping with A + tRNA are NAD5 and NAD4. COX2 stops with T + tRNA.

**Figure 1.**
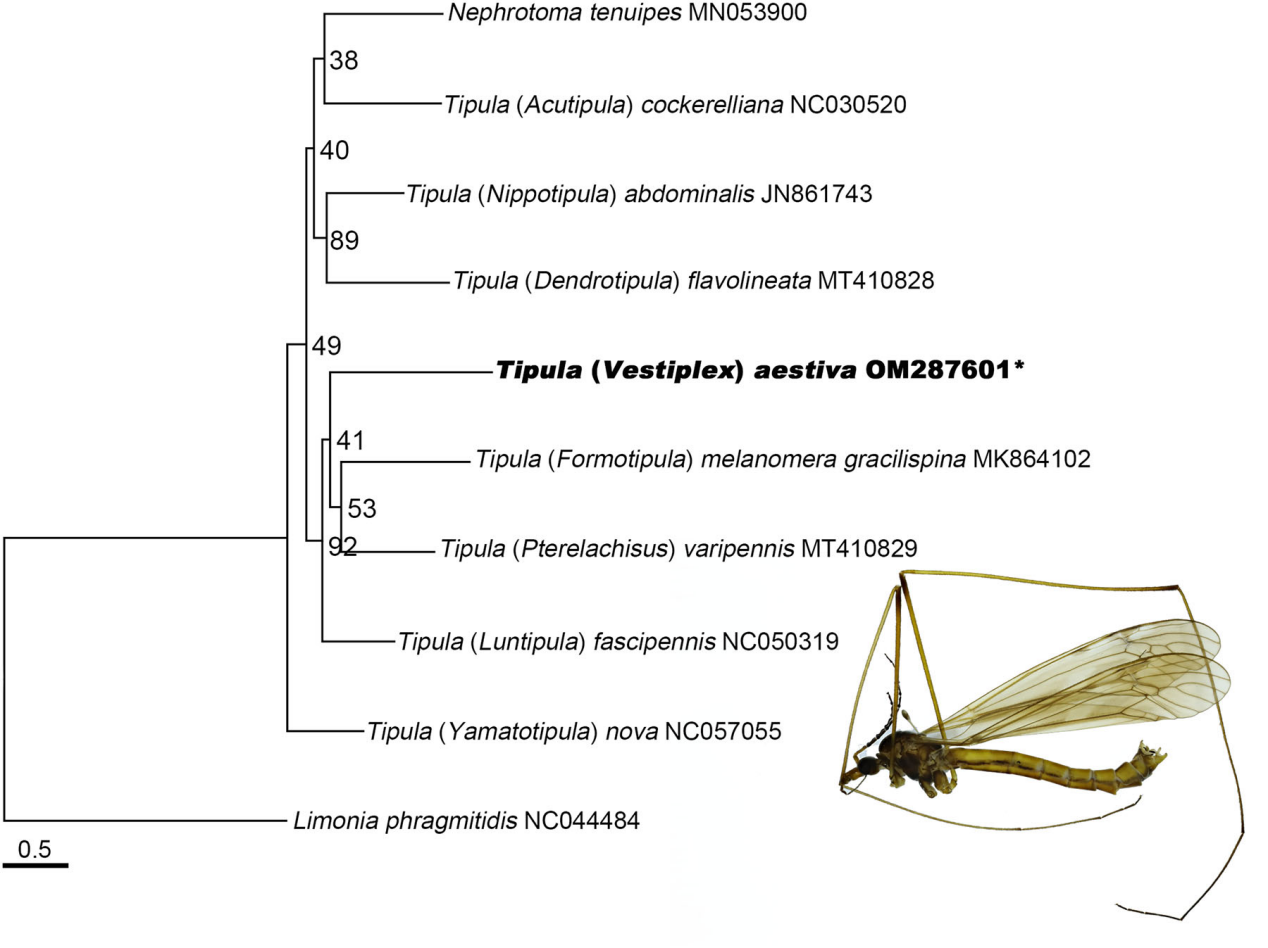
The maximum Likelihood phylogenetic tree of Tipulidae based on 13 PCGs with bootstrap values at the nodes.

In our research, the maximum likelihood (ML) analysis was conducted based on 13 PCGs from 9 species of the family Tipulidae (Ren et al. 2019b), and *Limonia phragmitidis* (Ren et al. 2019a) was chosen as outgroup using RAxML 8.2.12 (Stamatakis 2006). Our results support that the family Tipulidae is monophyletic (Figure 1). The monophyly of the genus *Tipula* was not supported, which is consistent with the phylogenetic result of the previous research (Petersen et al. 2010). The genus *Nephrotoma* is the sister group of subgenus *Acutipula*. The subgenus *Vestiplex* is the sister group of subgenus *Pterelachisus* and subgenus *Formotipula*, which is similar to the proposal of Savchenko (1979).

## Data Availability

The annotated mitochondrial genome described here is available in GenBank under accession OM287601. Raw data used for assembling was available in the SRA under accession SRR17634812 (https://www.ncbi.nlm.nih.gov/sra/PRJNA795034). The associated BioProject and Bio-Sample numbers are PRJNA795034 and SAMN24625269, respectively.

## References

[CIT0001] Alexander CP. 1935. New or little-known Tipulidae from eastern Asia (Diptera). XXV Philipp J Sci. 57:81–148.

[CIT0002] Alexander CP, Byers GW. 1981. Tipulidae. In: McAlpine JF, Peterson BV, Shewell GE, Teskey HJ, Vockeroth JR, Wood DM, Curran CH, editors. Manual of Nearctic Diptera. Vol. 1: Biosystematics Research Institute, Ottawa, Ontario, Monograph. p. 153–190.

[CIT0003] Beckenbach AT. 2011. Mitochondrial genome sequences of Nematocera (lower diptera): evidence of rearrangement following a complete genome duplication in a winter crane fly. GBE. 4:89–101. 22155689 10.1093/gbe/evr131 PMC3269971

[CIT0004] Gelhaus JK. 1986. Larvae of the crane fly genus *Tipula* in North America (Diptera: tipulidae). Univ Kansas Sci Bull. 53:121–182.

[CIT0005] Oosterbroek P. Catalogue of the craneflies of the world. (Diptera, Tipuloidea, Pediciidae, Limoniidae, Cylindrotomidae, Tipulidae). 2022. [accessed 2022 Jan 15]. http://ccw.naturalis.nl/.

[CIT0006] Petersen MJ, Bertone MA, Wiegmann BM, Courtney GW. 2010. Phylogenetic synthesis of morphological and molecular data reveals new insights into the higher-level classification of Tipuloidea (Diptera). Syst Ent. 35(3):526–545.

[CIT0007] Ren J, Yang Q, Gao S, Pan Z, Chang W, Yang D. 2019a. The mitochondrial genome of *Limonia phragmitidis* (Diptera tipulidae). Mitochondrial DNA Part B. 4(1):719–720.

[CIT0008] Ren J, Yang Q, Gao S, Pan Z, Chang W, Yang D. 2019b. The mitochondrial genome of *Nephrotoma tenuipes* (Diptera tipulidae). Mitochondrial DNA B Resour. 4(2):3092–3093. 33365867 10.1080/23802359.2019.1667271 PMC7706615

[CIT0009] Savchenko EN. 1979. Phylogenie und Systematik der Tipulidae. Translated and revised by Br. Theowald and G. Theischinger. Tijdschr Ent. 122: 91–126.

[CIT0010] Stamatakis A. 2006. RAxML-VI-HPC: maximum likelihood-based phylogenetic analyses with thousands of taxa and mixed models. Bioinformatics. 22(21):2688–2690. 16928733 10.1093/bioinformatics/btl446

[CIT0011] Starkevich P, Saldaitis A, Men Q. 2019. Four new crane fly species of subgenus *Tipula* (*Vestiplex*) from China. Zootaxa. 4679(1):zootaxa.4679.1.4–86. 31715970 10.11646/zootaxa.4679.1.4

[CIT0012] Zhang B, Gao S, Cao Y, Chang W, Yang D. 2019. The mitochondrial genome of *Tipula* (*Formotipula*) *melanomera gracilispina* (Diptera: Tipulidae). Mitochondrial DNA Part B. 4(1):240–241.

[CIT0013] Zhao C, Qian X, Wang S, Li Y, Zhang X. 2019. The complete mitochondrial genome and phylogenetic analysis of *Tipula* (*Yamatotipula*) *nova* Walker, 1848 (Diptera Tipulidae) from Qingdao, Shandong, China. Mitochondrial DNA B Resour. 4(2):4211–4213. 33366387 10.1080/23802359.2019.1693305 PMC7707724

